# Tobacco crop rotation enhances the stability and complexity of microbial networks

**DOI:** 10.3389/fmicb.2024.1416256

**Published:** 2024-06-19

**Authors:** Huilin Yan, Shaolong Wu, Ping Li, Xin Jin, Dejun Shi, Danjia Tu, Wei-ai Zeng, Lin Tan

**Affiliations:** ^1^State Key Laboratory of Plateau Ecology and Agriculture, Qinghai University, Xining, Qinghai, China; ^2^Tobacco Company of Hunan Province, Changsha, Hunan, China; ^3^Academy of Agriculture and Forestry Science of Qinghai University, Xining, Qinghai, China; ^4^College of Agriculture and Animal Husbandry, Qinghai University, Xining, Qinghai, China; ^5^Qinghai Province Grassland Improvement Experimental Station, Gonghe, Qinghai, China; ^6^Changsha Tobacco Company of Hunan Province, Changsha, Hunan, China; ^7^College of Plant Protection, Hunan Agricultural University, Changsha, Hunan, China

**Keywords:** tobacco, soil microorganism, continuous cropping, rotational cropping, co-occurrence network

## Abstract

**Introduction:**

The effects of continuous cropping and rotation cropping, two important tobacco cultivation practices, on soil microbial communities at different stages remain unclear. Different planting patterns have been shown to influence soil physical and chemical properties, which in turn can affect the composition and diversity of soil microbial communities.

**Methods:**

In order to investigate the impact of different planting methods on soil microbial community structure, we selected two representative planting methods: continuous cropping (tobacco) and rotational cropping (tobacco-maize). These methods were chosen as the focal points of our research to explore the potential effects on soil microbial communities. High-throughput sequencing technology was employed to investigate the structure of soil microbial communities, as well as their relationships with soil environmental factors, by utilizing the 16S rRNA, ITS, and 18S genes. Furthermore, the interaction among microorganisms was explored through the application of the Random Matrix Theory (RMT) molecular ecological network approach.

**Results:**

There was no significant difference in α diversity, but significant difference in β diversity based on Jaccard distance test. Compared to continuous cropping, crop rotation significantly increased the abundance of beneficial prokaryotes Verrucomicrobia and Rhodanobacter. These findings indicate that crop rotation promotes the enrichment of Verrucomicrobia and Rhodanobacter in the soil microbial community. AP and NH_4_-N had a greater effect on the community structure of prokaryotes and fungi in tobacco soil, while only AP had a greater effect on the community structure of protist. Molecular ecological network analysis showed that the network robustness and Cohesion of rotation were significantly higher than that of continuous cropping, indicating that the complexity and stability of molecular ecological networks were higher in the rotational, and the microbial communities cooperated more effectively, and the community structure was more stable.

**Discussion:**

From this point of view, rotational cropping is more conducive to changing the composition of soil microbial community, enhancing the stability of microbial network structure, and enhancing the potential ecological functions in soil.

## 1 Introduction

Tobacco (*Nicotiana tabacum L*.) is an economically important crop in China, primarily valued for its quality and yield (Zhou et al., 2020). Studies have shown that long-term continuous cropping causes soil problems such as shallow soil layer, structural damage, soil degradation, soil nutrient imbalance and soil-borne diseases, which affect the normal growth and development of tobacco and reduce the yield and quality of flue-cured tobacco (Tang et al., 2020). The yield and quality of tobacco leaves are mainly influenced by flue-cured tobacco varieties, ecological environment and cultivation techniques (Yang et al., 2020). In recent years, researchers have carried out a lot of studies from the perspective of adjusting tobacco planting patterns. The mechanism of alleviating and eliminating continuous cropping obstacles was discussed from the aspects of soil physical and chemical properties, soil microbial community structure, allelopathic and autotoxic effects of tobacco, etc., in order to provide reference for the prevention and control of continuous cropping obstacles and the sustainable and stable development of tobacco agriculture (Jiang et al., 2022; Jin et al., 2024). Continuous cropping refers to the continuous cultivation of the same crop on the same field in 1 year or 2 years. Rotational cropping refers to the cultivation of different crops or multiple cropping combinations on the same field in order between seasons and years (Ma et al., 2023). Through research and comparison, our experiment selected maize and corn bulk food crops with less labor, high mechanization and wide sales channels as continuous cropping and rotational cropping and implemented the planting mode of “tobacco-based and synergistic development of tobacco-grain industry”.

Soil health is the central theme of sustainable agricultural development. Soil microorganisms participate in soil ecological function, environmental function and immune function to drive the operation of soil life system, which is the core and key to maintain soil health (Wu et al., 2024). As an important component of soil ecosystem, microorganisms are extremely sensitive to environmental changes and are the core of soil reconfiguration. The interactions between plant-soil microbes are important for the functioning of terrestrial ecosystems and their response to global climate change. Soil microorganisms can directly interact with crops, such as through mycorrhizal symbiosis and rhizobia symbiosis to promote plant growth. Studies have shown that different crops can positively affect plant development and crop production by changing rhizosphere secretions to select or recruit soil microbiomes favorable to them (de Vries et al., 2020). Soil microbiome varies under different planting patterns. It was found that the rotation pattern could increase the number of colonies such as bacteria and actinomycetes in the soil, enrich the microbial population, increase the ratio of bacteria and fungi, and improve the community structure (Banerjee and van der Heijden, 2023). In general, as the number of soil bacteria and actinomycetes increases and the number of fungi decreases, the incidence of disease of crops grown in the soil will decrease. Existing study used PCR-RFLP technology to study and found that the bacterial community richness index and Shannon-wiener of rotating tobacco soil were higher than those of continuous cropping, indicating that the microbial community in rotating soil was relatively rich (Zheng et al., 2020). In addition, Niu et al found that the tobacco-maize crop rotation pattern could significantly reduce the incidence of virus disease (Niu et al., 2016). Rotational cropping can improve the quantity, variety and stability of soil microbial community, improve crop rhizosphere microenvironment, and reduce disease incidence, thus improving soil quality, crop yield and quality (Chen et al., 2021). Waha et al. (2020) showed that rotational planting of flue-soybean could significantly increase the number of bacteria, actinomycetes and some functional microorganisms related to soil nitrogen metabolism in crop rhizosphere soil, reduce the number of fungi, thereby reducing the incidence of soil-borne diseases and improving the metabolic cycle of soil. However, while significant progress has been made in understanding how soil microbes differ in rotational and continuous cropping compared to continuous cropping, much remains unknown about how microbes differ in both modes.

Ecological network refers to a topological model that simulates and builds biogeochemical cycles and energy flows in an ecosystem (Keyes et al., 2021). Molecular ecological network analysis is an emerging exploratory data analysis method, and ecological network analysis refers to the method of analysis and induction of ecological networks and the system theory of research (Deng et al., 2012). This method is based on random matrix theory and gene sequencing technology. Visualize microbial interactions and microbial system stability (Meyer et al., 2020). In recent years, ecological network analysis has been widely used in biological analysis, such as population relationship network, protein interaction network and gene regulation network, and has become a new research hotspot in ecology, especially microbial ecology (Zhou et al., 2010; Yuan et al., 2021). Molecular ecological network analysis is not only an important means to analyze the interactions between soil microorganisms and their influencing factors, but also to reveal the stability and complexity of soil ecological communities (Hernandez et al., 2021; Yuan et al., 2021). The stability and complexity of a network are often represented by robustness and Cohesion. Robustness refers to the ability of a microbial community to maintain its original structure and function after external interference (Yan et al., 2022). Cohesion quantifies the degree to which microbial communities are connected (Hernandez et al., 2021). It has also been applied to study the potential of soil resistance to continuous cropping obstacles (Tan et al., 2021). Therefore, our study used network analysis method to analyze soil-microbial interaction mechanism under tobacco continuous cropping and tobacco-maize rotational, so as to provide information for sustainable agricultural development.

The aim of this study was to elucidate the characteristics of soil prokaryotes, fungi and protists under continuous cropping and rotational cropping patterns of tobacco. To achieve the following goals: (i) the physical and chemical characteristics of Soil pH, total organic carbon (TOC), soil organic matter (SOM), total nitrogen (TN), total phosphorus (TP), total potassium (TK), nitrate nitrogen (NO_3_- N), ammonia nitrogen (NH_4_+-N), available phosphorus (AP), available potassium (AK), as well as the structure of soil bacteria and fungi community were analyzed and compared between the two types of soil. (ii) The relationship between environmental factors and microorganisms was analyzed, and the indicator species of soil prokaryotes, fungi and protists communities were identified in random forests under both models; (iii) Use of molecular ecological network analysis to reveal linkages between prokaryotes, fungi, and protist communities. By achieving the above research objectives, we hope to provide a new strategy and theoretical basis for tobacco continuous cropping and crop rotation.

## 2 Materials and methods

### 2.1 Study sites and sampling

The study site was located in Wenshan Zhuang and Miao Autonomous Prefecture (23° 15′N, 104° 35′E), Yunnan-Guizhou Plateau, Yunnan Province, China. It is a subtropical climate, 1,186 meters above sea level, the average temperature is 19°C, the frost-free period is 356 days, the sunshine is 2,228.9 h, and the average annual rainfall is about 779 mm. According to the classification of the Food and Agriculture Organization of the United Nations (FAO), the predominant soil type in the study region is Calcareous soil. For this study, we selected a total of eight tobacco growing areas with two different planting patterns: continuous cropping (C) and rotational cropping (R) involving both tobacco and maize. The planting is scheduled to take place in late July 2021. These selected areas will serve as the experimental sites to assess the impact of these planting methods on soil microbial communities. The same agronomic management was applied to all test plots. Soil samples were then collected by random sampling from multiple and rotating tobacco test plots. A soil drill with an inner diameter of 5 cm was used to collect 0 to 20 cm of tobacco soil at each point. Multiple soil samples (n = 5) were randomly collected at different locations in each plot, covering 0–20 cm soil layer. These samples are then combined to create a composite sample that represents each plot. A total of 19 composite soil samples were collected (10 continuous cropping soil samples and 9 rotating soil samples). Part of the tobacco soil collected was used for the detection of soil physical and chemical properties, and the other part was stored in the ice box and brought back to the laboratory at −80°C for DNA extraction and sequencing, and further analysis.

### 2.2 Soil physicochemical analyses

Soil pH, total organic carbon (TOC), soil organic matter (SOM), total nitrogen (TN), total phosphorus (TP), total potassium (TK), nitrate nitrogen (NO_3_- N), ammonia nitrogen (NH_4_+-N), available phosphorus (AP), available potassium (AK) and other parameters were determined according to the method adopted by Du et al. (2022) for the determination of soil physical and chemical properties. Physical and chemical properties of tobacco soil were determined at the Institute of Soil Science, Chinese Academy of Sciences, Nanjing, China. This method guarantees the accuracy and consistency of the obtained results.

### 2.3 DNA extraction, amplification, sequencing, and sequence analysis

Total DNA was extracted from 0.5 g tobacco soil in duplicate according to the manufacturer's instructions for the FastDNA™ SPIN kit (MoBio Laboratories, Carlsbad, CA). NanoDrop2000 spectrophotometer (Thermo) was used to determine the DNA quality and concentration. The concentration was above 20 ng/μL, and the A260/A280 was 1.8~2.0. Prokaryotic 16S rRNA gene was amplified using universal primers 515F (5′-GTGCCAGCMGCCGCGGTAA-3′) and 806R (5′-GGACTACHVGGGTWTCTAAT-3′). The polymerase chain reaction (PCR) amplification method follows the protocol of previous studies (Yan et al., 2022). ITS genes using universal primers 5.8F-Fun (5′-AACTTTYRRCAAYGGATCWCT-3′) and ITS4r-Fun (5′- AGCCTCCGCTTATTGATATGCTTAART-3′) amplification (Taylor et al., 2016). The 18S gene of the protoplasm (Step 1 primers): 615F: 5′- AGTGTCGATTCGGTTAAAARGCTCGTAGTYG-3′, 963R:5′-AAGATCGTACTGAAGARGAYATCCTTGGTG-3′; The second step of primers: 615 F 5′- AGTGTCGATTCGGTTAAAARGCTCGTAGTYG-3′, 947R: 5′-AAGARGAYATCCTTGGTG-3′ was amplified with a universal primer and supplemented with a sample specific barcode (Zhou et al., 2021). The thermal cycle conditions of PCR amplification were as follows:94°C for 3 min, 45 cycles, 94°C for 20 s, 57°C for 25 s, 72°C for 45 s, and finally 72°C for 10 min. PCR positive amplicon was detected and purified by 1% agarose gel electrophoresis and purified by kit (D2500-02, OMEGA BioTek). The purified amplicon was quantified by NanoDrop2000 spectrophotometer. The sample concentrations were all above 20 ng/μL, and the A260/280 ratio was 1.8-2.0. The sample was then mixed in equal proportions (150 ng) using a qubit fluorometer (Life technologies Holdings Pte Ltd, Singapore). The library was constructed using the VAHTS™ Nano DNA library Prep Kit for Illumina^®^ (Vazyme Biotech Co., Ltd, Nanjing, China) according to the reference instructions. The samples were gathered together and sequenced on the Hiseq sequencing machine (Illumina) at Magingene Biotechnology Co., LTD (Guangzhou, China).

### 2.4 Sequence data preprocessing and bioinformatics approaches

Fifty seven samples (19 × 3 community) of the original 16 s rRNA genes, ITS and 18S gene fragment sequencing data using integrate bioinformatics tools the internal pipeline (http://mem.rcees.ac.cn:8080) for processing (Feng et al., 2017). First, after the barcode is detected, the sequence is assigned to a single sample (sample sorting). After removing the barcode and primer sequence, the opposite sequence of the 16S rRNA gene was incorporated using a Flash program (Kong, 2011). For ITS sequences, the forward and reverse primers are removed, leaving the target sequence (Li S. et al., 2022). Subsequently, ITS flanking regions and non-fungal sequences were removed by an ITSx procedure. Next, Unoise3 algorithm (removing OTUs with abundance less than 8 sequences) was used to cluster the sequences into operational taxa (OTUs), generating a zero-radius OTU table (Edgar, 2016). Based on RDP (training set No. 18, July 2020) and warcup database V2 published in June 2016, and PR2 database (version_4.10.0_mothur) (Guillou et al., 2012), the classification information of prokaryotes, ITS and 18S was labeled. Finally, 71,594, 23,114 and 87833 zOTUs were obtained in prokaryotes, ITS and 18S, respectively, which were then used for downstream analysis.

### 2.5 Statistical analysis

This study adopts open analysis pipeline (http://mem.rcees.ac.cn:8080) calculation richness index to evaluate multiple cropping and rotation of the microbial community. Abundance is obtained by calculating the number of observed species shown in the zOTUs table. Nonmetric multidimensional scaling analysis (NMDS) was used to analyze the β-diversity of microbial communities in multiple cropping and rotation soils. Jaccard distance and difference tests based on multiple response displacement process (MRPP), one-way analysis of variance (ANOSIM) and multiple analysis of variance of displacement (PERMANOVA) were used. Significant differences were determined by Tukey post hoc tests and LSD tests, and community structure under the two planting methods was compared (Anderson, 2001). The Random Forests (RF) analysis method was used to further explore the markers of significant differences in soil microbial communities under multiple cropping and rotational cropping, and analyze the differences in the abundance of soil microbial communities under different treatments (Statnikov et al., 2008).

### 2.6 Random matrix theory based molecular ecology networks and analysis

Cropping and crop rotation mode in order to reveal the relationship between microbial community, we use a publicly available pipe network (http://mem.rcees.ac.cn:8081) constructed molecular ecology (Feng et al., 2022). Based on the stochastic matrix theory (RMT) method, in-domain ecological network (MEN) was constructed. We performed Spearman correlation screening on RMT results of microbial communities, and used thresholds *r* ≥ 0.95, 0.86 for prokaryotes, fungi and protists, respectively. To assess the importance of each observed MEN, we used the Maslov-Sneppen method to generate 100 randomly reconnected networks (Bascompte et al., 2003) and performed topological property checks for each index. Based on the values of intra-module connections (Zi) and inter-module connections (Pi), we classify nodes into four categories: peripherals, connectors, module hubs, and network hubs (Olesen et al., 2007). Module hubs, connectors and network hubs are considered as key species in molecular ecological networks. At the same time, in order to prove whether and how the two planting methods affect the stability of the network, the robustness and cohesion of the network are calculated. Robustness is defined as the proportion of remaining species in the network after random removal of 50% of nodes (Deng et al., 2012; Montesinos-Navarro et al., 2017). We calculate two cohesion values (positive and negative) based on ptwo correlation. This is a measure of the degree of relevance of cooperative behavior or competitive interactions (Herren and McMahon, 2017), which can indirectly represent complexity. Positive correlations can indicate facilitative/supportive relationships between taxa and reflect ecological or functional similarities (Barberán et al., 2012; Durán et al., 2018), while the negative correlation may indicate that competition reflects different niche needs among taxa (Zelezniak et al., 2015). Using Gephi (v0.9.2; https://gephi.org/) Visualizing the network.

## 3 Results

### 3.1 Effects of continuous and rotational cropping on soil physicochemical properties

Table 1 presents the soil physicochemical properties comparing continuous and rotational cropping. The activities of total organic carbon (TOC), soil organic matter (SOM), total nitrogen (TN), total potassium (TK), nitrate nitrogen (NO_3_- N), and ammonium nitrogen (NH_4_+ N) were significantly higher in rotational cropping compared to continuous cropping (*P* < 0.05). However, there was no significant difference observed in soil pH and total phosphorus (TP) between the two planting patterns.

**Table 1:** Under continuous and rotational cropping systems, the soil physicochemical properties were evaluated.

	**Continuous cropping**	**Rotational cropping**
Total organic carbon (TOC g/kg)	13.93 ± 2.22 a	16.74 ± 1.76 b
Soil organic matter (SOM g/kg)	24.72 ± 3.97 a	28.20 ± 2.99 b
Total nitrogen (TN g/kg)	1.34 ± 0.17 a	1.62 ± 0.17 b
Total phosphorus (TP g/kg)	1.06 ± 0.23 a	1.23 ± 0.16 a
Total potassium (TK g/kg)	23.65 ± 2.94 a	30.29 ± 3.03 b
Nitrate nitrogen (NO_3_- N mg/kg)	10.61 ± 4.53 a	23.01 ± 13.19 b
Ammonia nitrogen (NH_4_+-N mg/kg)	3.77 ± 0.57 a	7.74 ± 3.36 b
Available phosphorus (AP mg/kg)	63.72 ± 16.73 a	101.50 ± 27.07 b
Available potassium (AK mg/kg)	582.79 ± 65.51 a	713.58 ± 88.50 b
pH	6.23 ± 0.32 a	6.09 ± 0.38 a

One-way ANOVA was conducted to determine significant differences between treatments, with different letters within the same column indicating statistically significant variations (P < 0.05). a and b represent the significance between the two groups.

**Table 2:** Properties of empirical and randomized molecular ecology networks (MENs) for prokaryotic communities under continuous and rotational cropping.

**Group**	**Molecular ecological network**											**Random network**		
	**Similarity threshold**	**Nodes**	**Links**	**Average degree (avgK)**	**Average clustering coefficient (avgCC)**	**Average path distance (GD)**	**Centrali- zation of degree (CD)**	**Density (D)**	**Transitivity (Trans)**	**Modularity**	**R** ^2^	**Average clustering coefficient (avgCC)**	**Average path distance (GD)**	**Modularity**
**C_16S**	0.810	657	17,299	52.661	0.467^a^	3.109^b^	0.163	0.080	0.525	0.269^c^	0.642	0.229 ± 0.029	2.276 ± 0.083	0.093 ± 0.017
**R_16S**	0.810	862	6,673	15.482	0.359^a^	4.109^b^	0.066	0.017	0.405	0.522^c^	0.618	0.522 ± 0.054	2.872 ± 0.128	0.210 ± 0.031
**C_ITS**	0.800	60	74	2.466	0.265^a^	4.322^b^	0.110	0.041	0.375	0.741^c^	0.822	0.550 ± 0.038	2.827 ± 0.128	0.089 ± 0.006
**R_ITS**	0.800	104	129	2.480	0.176^a^	4.982^b^	0.073	0.024	0.494	0.741^c^	0.981	0.470 ± 0.020	1.418 ± 0.001	0.067 ± 0.003
**C_18S**	0.860	239	423	3.539	0.292^a^	5.067^b^	0.073	0.014	0.432	0.728^c^	0.943	0.416 ± 0.007	1.336 ±0.001	0.051 ± 0.003
**R_18S**	0.860	318	584	3.672	0.269^a^	6.270^b^	0.029	0.011	0.323	0.756^c^	0.790	0.417 ± 0.005	1.31. ± 0.001	0.051 ± 0.002

Randomized networks were performed by rewiring all the nodes and links corresponding to empirical networks 100 times. C, continuous cropping; R, rotational cropping.

^a^Significant difference in avgCC between empirical and randomized networks based on Student's t-test.

^b^Significant difference in GD between empirical and randomized networks based on Student's t-test.

^c^Significant difference in M between empirical and randomized networks based on Student's t-test.

### 3.2 Characteristics of soil microbial communities

#### 3.2.1 Microbial community structure

Microbial community alpha-diversity was assessed using Chao1 and richness indices, while evenness was evaluated using the Shannon index. The findings indicated that there was no significant difference in α-diversity between continuous cropping and rotational cropping (Supplementary Figure S1).

To investigate the disparities in soil microbial communities between continuous and rotational cropping, we employed the Jaccard dissimilarity-based non-metric multidimensional scaling (NMDS) technique to visualize variations in group structure. The results revealed significant differences in community structure among prokaryotes (stress = 0.08), fungi (stress = 0.103), and protists (stress = 0.073) between continuous and rotational cropping systems (Supplementary Figure S2). The analysis of the results demonstrated significant disparities in the composition and structure of soil microbial communities between continuous cropping and rotational cropping systems. Different tests such as MRPP, ANOSIM and PERMANOVA confirmed that there were significant differences in prokaryotic communities between continuous cropping and rotation (MRPP, P = 0.003; ANOSIM, P = 0.004; PERMANOVA, P = 0.012). There were significant differences in ITS communities (MRPP, *P* = 0.002; ANOSIM, *P* = 0.001; PERMANOVA, *P* = 0.001). There were significant differences in 18S communities (MRPP, *P* = 0.027; ANOSIM, *P* = 0.013; PERMANOVA, *P* = 0.021) (Supplementary Table S1).

At the phyla level, classification was conducted to determine the relative abundance of taxa for all three microbiota. Whether in continuous or rotational cropping, the dominant phyla of prokaryotes in the soil were primarily composed of Proteobacteria (C: 40.05%, R: 41.64%), Acidobacteria (C: 13.83%, R: 12.87%), Actinobacteria (C: 11.26%, R: 11.14%), Gemmatimonadetes (C: 6.59%, R: 6.80%), Bacteroidetes (C: 6.01%, R: 6.68%), and Chloroflexi (C: 5.25%, R: 5.40%) (Supplementary Figure S3). Among them, Verrucomicrobia, a non-dominant bacterium, exhibited a significant difference between rotational and multiple cropping (P < 0.005). The dominant phyla of fungi in the soil primarily consisted of Ascomycota (C: 52.75%, R: 44.71%), Basidiomycota (C: 27.49%, R: 34.53%), Unclassified (C: 16.76%, R: 15.56%), Zygomycota (C: 1.63%, R: 3.14%), and Chytridiomycota (C: 1.34%, R: 2.05%). The dominant phyla of protists in the soil were mainly composed of Archaeplastida (C: 45.90%, R: 70.35%), Opisthokonta (C: 25.81%, R: 15.11%), Rhizaria (C: 11.85%, R: 5.68%), Stramenopiles (C: 5.22%, R: 3.30%), and Amoebozoa (C: 5.01%, R: 2.18%).

At the genus level, the dominant prokaryotic genus in soil were mainly composed of *Sphingomonas* (C: 7.08%, R: 7.56%), *Gemmatirosa* (C: 4.62%, R: 4.77%), *Saccharibacteria genera incertae sedis* (M: 4.28%, R: 4.91%), *Bryobacter* (C: 2.61%, R: 2.67%), Gp1 (C: 2.60%, R: 2.32%), *Rhodanobacter* (C: 0.78%, R: 2.61%), *Candidatus Solibacter* (C: 1.68%, R: 0.77%), *Ramlibacter* (C: 0.61%, R: 1.32%) significant differences in relative abundance of *Candidatus Solibacter, Sphingomonas, Rhodanobacter* and *Ramlibacter* (ANOVA, P < 0.005). The dominant fungi genus in soil were mainly composed of *Unclassified* (C: 39.38%, R: 59.63%), *Coprinellus* (C: 10.89%, R: 7.13%), *Hypocrea* (C: 8.89%, R: 1.09%), *Exophiala* (C: 0.18%, R: 9.47%), *Trechispora* (C: 8.39%, R: 0.17%), the non-dominant strain *Metacordyceps* showed significant difference between the two planting patterns (P < 0.005). The dominant protist genus in soil were mainly composed of *Embryophyceae XX* (C: 44.04%, R: 69.38%), *Arachnida* (C: 4.23%, R: 2.49%), *Annelida XX* (C: 4.48%, R: 0.31%), *Enoplea X* (C: 3.02%, R: 0.85%), *Catenulida* (C: 3.57%, R: 0.02%), *Leptophryidae* (C: 2.71%, R: 0.30%) (Supplementary Figure S3).

#### 3.2.2 Differential distribution of soil microbial communities under continuous and rotational cropping

**Figure 1:**
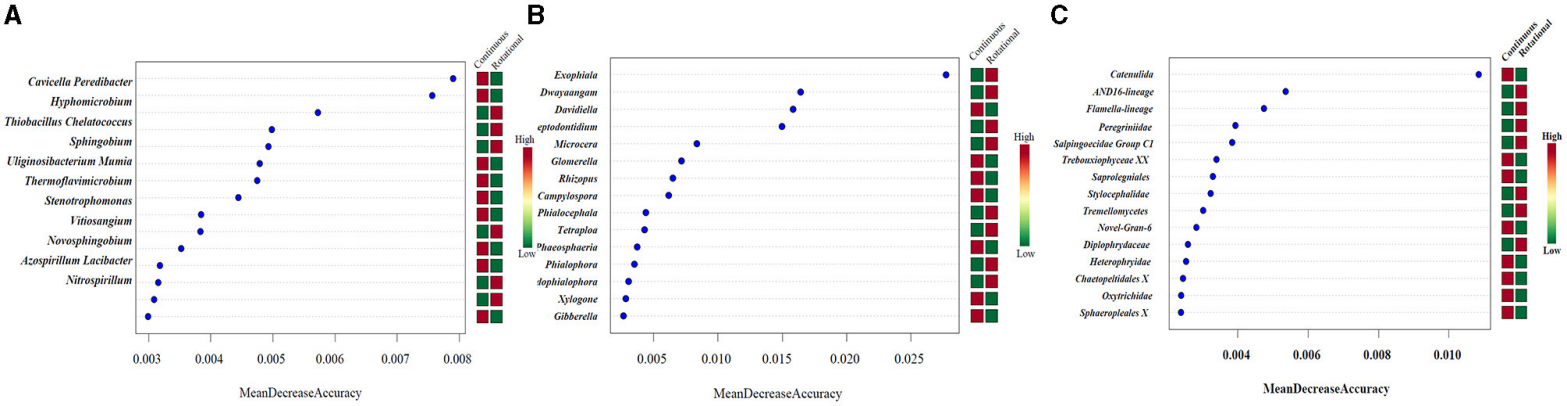
Random forest analysis showed the differential distribution of soil microbial communities under different planting patterns. **(A)** Prokaryotes. **(B)** ITS. **(C)** 18S.

The aforementioned analysis provides insights into the potential impacts of different planting patterns on soil microbial community structure and diversity. To delve deeper into the specific effects of these planting patterns on the composition of soil microbial communities in tobacco, we employed random forest tools to identify the most distinguishable genera between continuous and rotational cropping systems. The 11 genera with the greatest differences in the distribution of prokaryotes in Figure 1A. The 15 genera with the greatest differences in the distribution of fungi in Figure 1B. The 15 genera with the greatest differences in the distribution of protist in Figure 1C. It is worth noting that all the biomarkers identified under continuous and rotational cropping were significantly different at the genus level. Significant differences in relative abundance of Candidatus Solibacter and Ramlibacter (*P* < 0.005). Significant differences in relative abundance of Embryophyceae XX, Catenulida and Peronosporales (*P* < 0.005).

#### 3.2.3 The relationship between soil microbial and soil physicochemical properties

**Figure 2:**
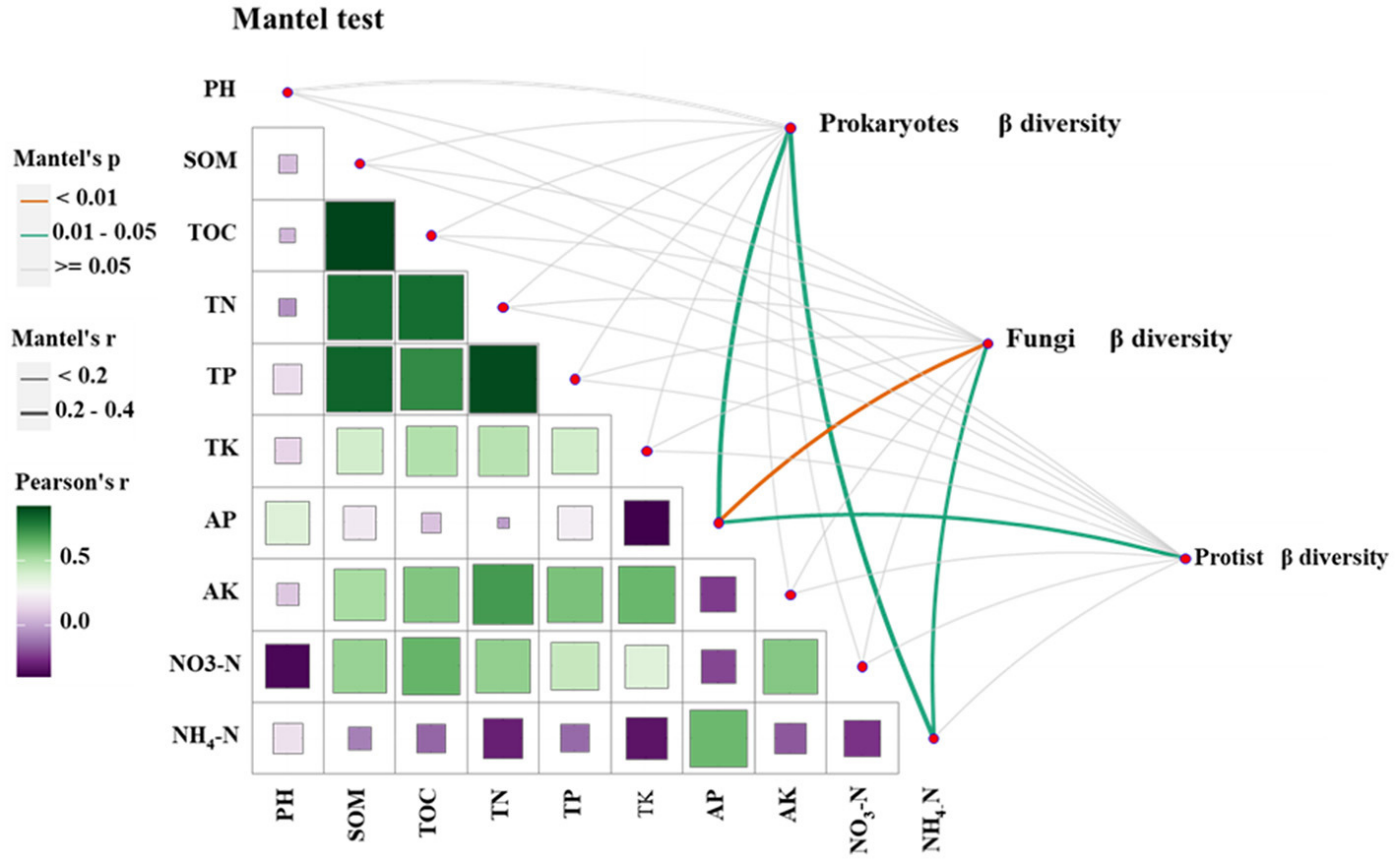
The relationship between microbial community and soil physicochemical properties was analyzed by Mantel test.

To investigate the variation in the influence of different environmental factors on the community structure of soil prokaryotes, fungi, and protists, a Mantel test was conducted. Correlation analyses were performed on the Bray-Curtis and Jaccard distance matrices of soil prokaryotes, fungi, and protist communities from continuous and rotational cropping systems. The findings revealed that AP (available phosphorus) and NH_4_-N (ammonium nitrogen) had a more pronounced impact on the community structure of tobacco soil prokaryotes and fungi. However, only AP exhibited a greater effect on the protist community structure (Figure 2).

### 3.3 Effects of continuous and rotational cropping on soil microbial community interaction and stability

**Figure 3:**
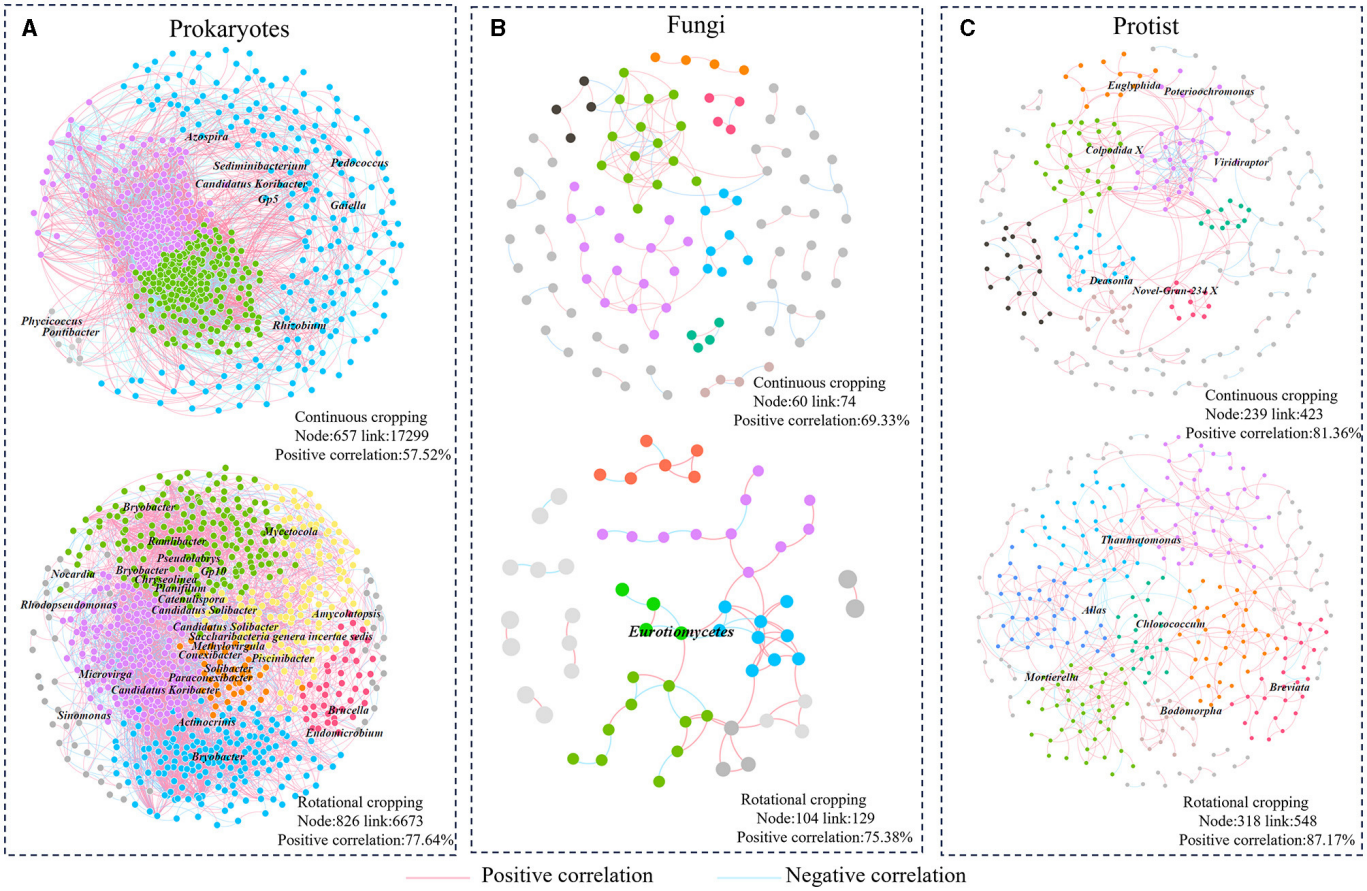
Analysis of community structure network of prokaryotes, fungi and protists. **(A)** Prokaryotic networks. **(B)** Fungal networks. **(C)** Protist networks. Different colors represent different modules, and modules with <5 nodes are gray.

To investigate the disparities in microbial community interactions between continuous cropping and rotational cropping systems, we constructed six molecular ecological networks (MENs) based on the RMT (Random Matrix Theory) approach. The MENs were constructed using the sequencing data of 16S rRNA, ITS, and 18S to capture the interactions within each treatment. To ensure comparability across different networks, we established MENs for the soil samples under multiple cropping and rotation using consistent thresholds. Specifically, thresholds of 0.81 were applied for prokaryotic communities, 0.80 for fungi communities, and 0.86 for protist communities. The topological characteristics of the molecular ecological networks were then analyzed. The average path length (GD) for each network ranged from 3.109 to 6.270. These values closely approximated the logarithm of the total number of nodes in the network and were higher than those observed in the corresponding random networks. This shows that MENs has typical small-world network characteristics. The number of nodes in the communities of prokaryotes, fungi and protists in rotation was higher than that in continuous species (prokaryotes: C:657 R:862; fungi: C:60 R:014; protist: C:239 R:318). Except for prokaryotes, the links of fungi and protist communities in rotation were more than those in continuous cropping (fungi: C:74 R:129; protist: C: 423 R:548), indicating that the network was more complex and the connections between microbiota were closer under rotational cropping. The positive correlation ratio of prokaryotes (77.64%), fungi (75.38%) and protists (87.17%) in rotational cropping network was higher than that of continuous cropping prokaryotes (57.52%), fungi (69.33%) and protists (81.36%) (Figure 3), indicating that rotational cropping could strengthen the cooperative relationship among microorganisms.

**Figure 4:**
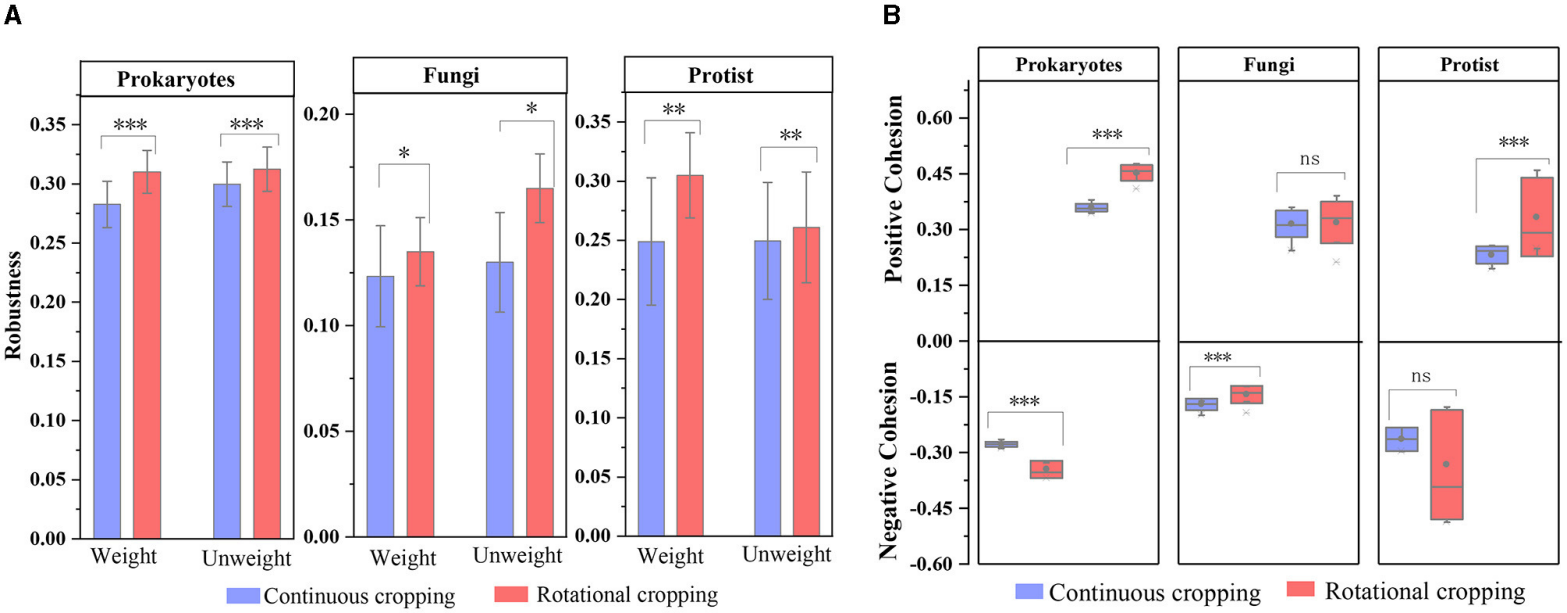
The complexity and stability of soil microbial community. **(A)** Cohesion analysis of the microbial communities, where blue represents continuous cropping samples and the red represents rotational cropping samples. **(B)** Robustness analysis of the microbial communities. The levels of significance are indicated as 0.001***, 0.01**, and 0.05*.

In addition, we calculated the robustness and Cohesion values of each network, which represent the stability of the network. Under continuous cropping (C) and rotation (R), C (average 0.282 ± 0.019) was lower than R (average 0.310 ± 0.018) in prokaryotic community, and C (average 0.123 ± 0.016) was lower than R (average 0.135 ± 0.023) in fungi community. In the protist community, C (average 0.249 ± 0.053) was lower than R (average 0.305 ± 0.036), indicating that the network had higher stability under rotational cropping (Figure 4A). The Cohesion analysis showed that the absolute value of negative Cohesion of prokaryotes and protists was higher in continuous cropping soils, indicating that the competition between prokaryotes and protists was more intense than that in rotational soils (Figure 4B). Conversely, rotational soils showed a higher absolute value positive Cohesion for fungi communities, indicating increased cooperation between fungi compared to continuous cropping soils.

## 4 Discussion

Continuous cropping is a prominent practice in Chinese agriculture, representing an important form of intensive cultivation (Zhao et al., 2016). However, the expansion of continuous cropping is constrained by various local factors, such as heat, soil conditions, water availability, fertilizer requirements, and labor availability (Waha et al., 2020). Due to the increased water and nutrient demands associated with continuous cropping, it is crucial to enhance farmland infrastructure and augment fertilizer application to support multi-cropping production (Gaba et al., 2015). On the other hand, rotational cropping involves the sequential planting of different crop combinations in the same field, either between seasons or across years (Barbieri et al., 2017). Research has demonstrated that rotating host and non-host crops can reduce the incidence of various crop diseases, such as tobacco black shank disease, broad bean root rot disease, and beet brown spot disease, by diminishing the pathogen population in the soil (Naseri and Hemmati, 2017; Gai et al., 2023; Tan et al., 2023).

### 4.1 Effects of two planting patterns on soil characteristics

The various planting patterns entail distinct nutrient management practices, leading to alterations in soil organic matter content and influencing the physicochemical properties of the soil. Consequently, these changes can impact the composition and diversity of soil microorganisms. Extensive research has demonstrated the effects of different planting patterns on soil physicochemical properties and microbial community structure (Xiao et al., 2023; Zhang M. et al., 2023). For instance, total organic carbon (TOC), soil organic matter (SOM), total nitrogen (TN), total potassium (TK), nitrate nitrogen (NO3-N), and ammonium nitrogen (NH4-N) were found to be significantly higher in rotational cropping compared to continuous cropping, which aligns with findings from previous studies (Li H. et al., 2022).

### 4.2 Effects of two planting patterns on soil microbial community composition

The quantity and composition of soil microorganisms, across the different groups the changes in soil nutrient dynamics, are significantly influenced by different crop planting methods. It has been well-documented that crop rotation has substantial effects on soil physical and chemical properties, which are closely associated with alterations in soil microbial community characteristics (Zhang H. et al., 2023). Significant differences were observed in the abundance of Verrucomicrobia at the phylum level. Verrucomicrobia is known for its ability to produce beneficial compounds such as antibiotics, which can aid in pathogen suppression. This phylum plays a crucial role in ecosystems by contributing to soil fertility enhancement, water quality improvement, and facilitating nutrient uptake by plants and animals. Moreover, Verrucomicrobia can contribute to the reduction of pollutant emissions and mitigate environmental damage caused by pollutants (Bünger et al., 2020; Larsson and Flach, 2022). Our findings indicate significant variations in the relative abundance of *Candidatus Solibacter, Sphingomonas*, and *Ramlibacter* (*P* < 0.005), previous studies have shown that *Candidatus Solibacter* can decompose organic matter, utilize carbon sources, and promote soil health (Wang et al., 2022). It was found that *Sphingomonas* cell membrane not only contains *sphingomolipids* which are more hydrophobic than lipopolysaccharides, but also has efficient metabolic regulation mechanism and gene regulation ability, which makes *sphingomonas* have great application potential in environmental remediation and promoting plant growth (de Vries et al., 2019). Rhodanobacter populations have also been shown to be antagonistic to the root rot fungal pathogen Fusarium solani and may be involved in nitrogen cycle processes (Huo et al., 2018). Our results further revealed a significant increase in the relative abundance of Peronosporales in continuous cropping compared to rotational cropping (*P* < 0.005). Peronosporales has been identified as a pathogenic group affecting numerous economically important plants, often leading to disease outbreaks and substantial losses, such as seedling wilting, white rust in rapeseed, and various downy mildew diseases (McGowan and Fitzpatrick, 2020). Based on these findings, we posit that rotational cropping may exhibit a more dominant role in mitigating tobacco root rot compared to continuous cropping.

### 4.3 Effects of soil physicochemical properties on the composition of the soil microbial community

Previous studies have provided evidence that different planting methods have the potential to modify soil physicochemical properties and influence the diversity and composition of microorganisms (Li et al., 2019). Numerous soil factors, including organic matter content, pH, salinity, among others, can contribute to alterations in soil microbial composition (Bastida et al., 2021). It is worth noting that different planting methods can lead to changes in these soil factors, further impacting the microbial communities present in the soil. Previous studies have shown that there is a significant correlation between microbial community and pH, and different microorganisms are very sensitive to environmental pH, and small changes will also cause changes in microbial community structure (Bastida et al., 2021; Naz et al., 2022). However, in our study, mental tests found no significant correlation between pH and microbial community composition, which is consistent with previous findings (Guo and Zhou, 2021). In our study, AP and NH_4_-N had a greater effect on the community structure of prokaryotes and fungi in tobacco soil, and only AP had a greater effect on the community structure, similar to previous studies (Zeng et al., 2017).

### 4.4 Effects of two planting patterns on soil microbial ecological network

In crop production activities, the ecological functions of soil will be different due to different planting methods (Bünger et al., 2020). Different planting methods can induce variations in microbial communities and their structures, thereby influencing diverse soil ecosystem functions. Within the intricate soil microbial ecosystem, microorganisms coexist and interact with each other within a complex network structure (Bastida et al., 2021). The results of this study (Figure 3) reveal the ecological characteristics of highly connected microorganisms in different structural modules of the soil microbial communities of the two planting styles. The community composition and diversity of prokaryotes, fungi and protists in rotational cropping were richer and more modular than that of continuous cropping. In molecular ecological networks, the number of nodes, the number of connections and the average connectivity are used to indicate the scale and complexity of the network, and the greater the average connectivity indicates the more complex connections between network nodes (Yuan et al., 2021). The higher number of nodes in crop rotation and the symbiotic relationship between species in the network indicated that the scale of soil microbial network in rotational cropping was larger and the relationship between species was more complex. The interrelationships between species in the network can be described as positive and negative correlations, with positive correlation indicating that the species have the same ecological niche or have symbiotic relationships, and negative correlation representing competition or predation relationships (Deng et al., 2012; Jiao et al., 2020). The number of network positive correlation connections of crop rotation was higher than that of continuous cropping, indicating that crop rotation enhanced the cooperation among soil microorganisms. It can be inferred that when disturbance occurs in the external environment, rotational cropping microbial network can slow down the transmission of disturbance and keep its structure stable, and stable microbial network may improve the participation in soil nutrient cycling, which is similar to the results of previous studies (Yan et al., 2022). In addition, we calculated the robustness and Cohesion of microbial networks to demonstrate the complexity and stability of molecular ecological networks. The results showed that the microbial communities had higher degree of cooperation, more effective interaction and more stable community structure under rotational cropping. Our results suggested those stronger interspecific interactions can enhance community stability. Closely symbiotic species in a community can greatly influence the stability and function of an ecosystem through aggregation modules (Wan et al., 2020; Li et al., 2021). Rotational cropping significantly stimulates dynamic responses to network complexity, which results in higher community stability, and therefore the associated ecosystem functions become stronger under rotational cropping cultivation. The enhancement of ecosystem functions may provide better service for forage growth and further increase the tobacco yield.

Community stability plays a crucial role in determining the ecological functioning of soil ecosystems. The stability of microbial communities is closely linked to their ability to resist and recover from disturbances, such as changes in environmental conditions or the introduction of new species. A stable microbial community contributes to enhanced nutrient cycling and availability, promoting plant growth and productivity. The diversity within a stable microbial network ensures the presence of a wide range of functional groups, each performing specific ecological functions. Several studies have demonstrated the positive correlation between community stability and ecosystem functioning. For example, research by Smith et al. (2016) found that a more stable microbial community in agricultural soils resulted in increased carbon and nitrogen cycling rates, highlighting the importance of stability in maintaining essential soil processes (Smith et al., 2016). Similarly, Johnson et al. (2020) observed that a stable microbial community contributed to improved nutrient availability and plant growth (Bender et al., 2016). Therefore, soil with rotation is more organized and diversified than that with continuous cropping, which enhances the ability of soil microorganisms to use nutrients and improves the microecology of cultivated land.

## 5 Conclusion

In this study, the soil microbial communities of two distinct cropping patterns in Yunnan, China, were investigated using Illumina HiSeq sequencing technology in conjunction with molecular ecological network analysis. The results revealed significant disparities in microbial β diversity between rotational cropping and continuous cropping. Notably, the abundance of beneficial prokaryotes, namely Verrucomicrobia and Rhodanobacter, was significantly higher in rotational cropping compared to continuous cropping. Furthermore, the community structure of prokaryotes and fungi in tobacco soil was significantly influenced by available phosphorus (AP) and ammonium nitrogen (NH_4_-N), while only AP exerted a significant impact on the protist community structure. Rotational cropping fostered increased interactions within soil prokaryotes, fungi, and protists, leading to more intricate network structures. Moreover, the network robustness and cohesion were significantly higher in the rotational cropping system compared to continuous cropping. Overall, these findings provide novel insights into the relationship between changes in soil physical and chemical properties and differences in microbial community dynamics under continuous cropping and crop rotation modes.

## Data availability statement

The original contributions presented in the study are publicly available. Data has been deposited in the China National Microbiology DataCenter (NMDC) with the accession number NMDC20165061. URL is https://nmdc.cn/resource/en/genomics/sample/detail/NMDC20165061.

## Author contributions

HY: Formal analysis, Visualization, Writing – original draft. SW: Methodology, Writing – review & editing. PL: Writing – review & editing. XJ: Formal analysis, Writing – review & editing. DS: Data curation, Writing – review & editing. DT: Data curation, Writing – review & editing. W-aZ: Methodology, Validation, Writing – review & editing. LT: Conceptualization, Writing – review & editing.
