# Supplementary material for: Tobacco crop rotation enhances the stability and complexity of microbial networks
Source: Front Microbiol. 2024 Jun 19;15:1416256. doi: 10.3389/fmicb.2024.1416256 (PMC11220274; doi:10.3389/fmicb.2024.1416256)

**Supplementary Material**

**Tobacco crop rotation enhances the stability and complexity of microbial networks**

Huilin Yan^1^, XuexiaWu^1^, Shaolong Wu^7^, Ping Li^3^, Xin Jin^2^, Dejun Shi^4^, Danjia Tu^4^, Wei-ai Zeng^6*^, Lin Tan^5^^*^

*^1^State Key Laboratory of Plateau Ecology and Agriculture, Qinghai University, Xining, Qinghai, China*

*^2^College of Agriculture and Animal Husbandry, Qinghai University, Xining, Qinghai 810016, China*

*^3^Academy of Agriculture and Forestry Science of Qinghai University, Qinghai, China*

*^4^Qinghai Province Grassland Improvement Experimental Station, Gonghe Qinghai, China*

*^5^College of Plant Protection, Hunan Agricultural University, Changsha, Hunan, China*

*^6^Changsha Tobacco Company of Hunan Province, Changsha, Hunan, China*

*^7^Tobacco Company of Hunan Province, Changsha, Hunan, China*

* Correspondence author

E-mail addresses: [zwa10537@163.com](mailto:slwuhnyc@126.com;) (Wei-ai Zeng) , hqltanlin@163.com (LinTan)

Detailed information of the Supplementary Material

The number of Tables: 1

The number of Figures: 3

Table S1 Dissimilarity test of soil microbial communities. C: Continuous cropping; R: Rotational cropping

| Community | Distance | Group | MRPP | | ANOSIM | | PERMANOVA | |
| --- | --- | --- | --- | --- | --- | --- | --- | --- |
|  |  |  | Delta | P | r | P | Pesudo-F | P |
| prokaryotic |  | C *V.S*. R | 0.5852 | **0.001** | 0.4639 | **0.001** | 2.1106 | **0.001** |
| ITS | Jaccard | C *V.S*. R | 0.7701 | **0.001** | 0.7064 | **0.001** | 2.1708 | **0.001** |
| 18S |  | C *V.S*. R | 0.6910 | **0.003** | 0.4330 | **0.001** | 2.1987 | **0.001** |
| prokaryotic |  | C *V.S*. R | 0.5265 | **0.003** | 0.2828 | **0.004** | 2.6187 | **0.012** |
| ITS | Bray-Curtis | C *V.S*. R | 0.8026 | **0.002** | 0.5475 | **0.001** | 2.9750 | **0.001** |
| 18S |  | C *V.S*. R | 0.5010 | **0.027** | 0.1706 | **0.013** | 3.1197 | **0.021** |

Figure S1 Two-tailed Mann-Whitney U test comparison of the α-diversity of the soil microbial communities under two planting patterns.


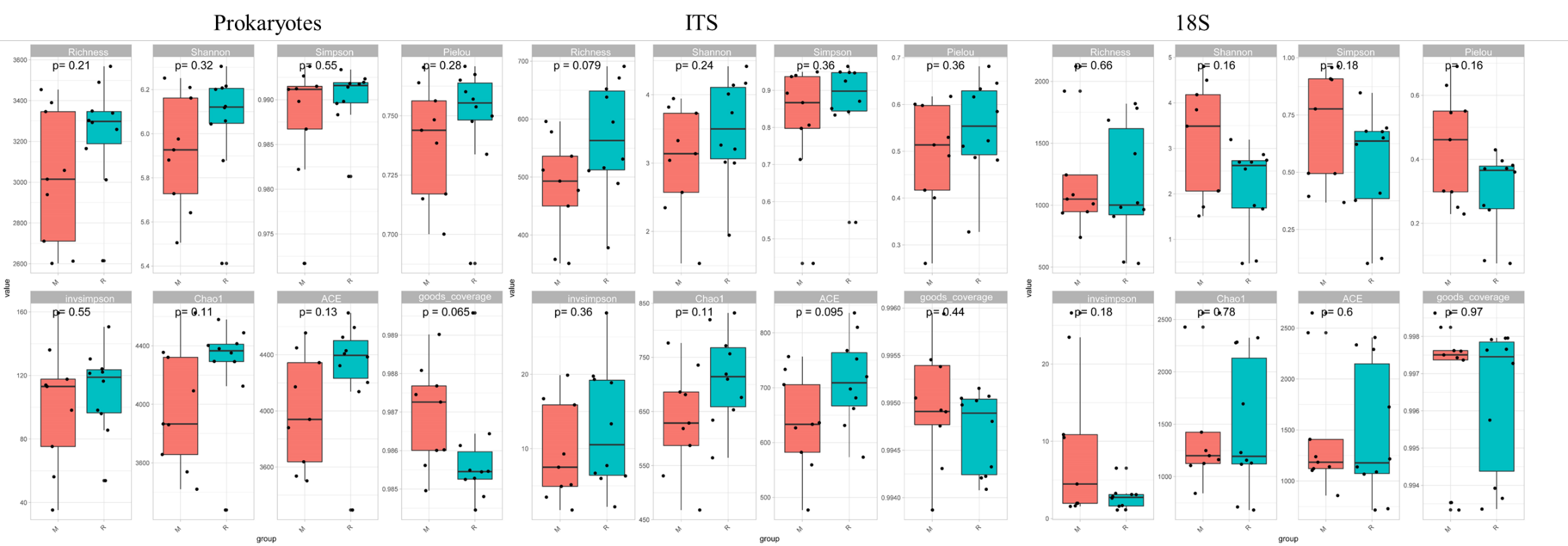


Figure S2 The NMDS analysis based on jaccard distance of microbial communities.


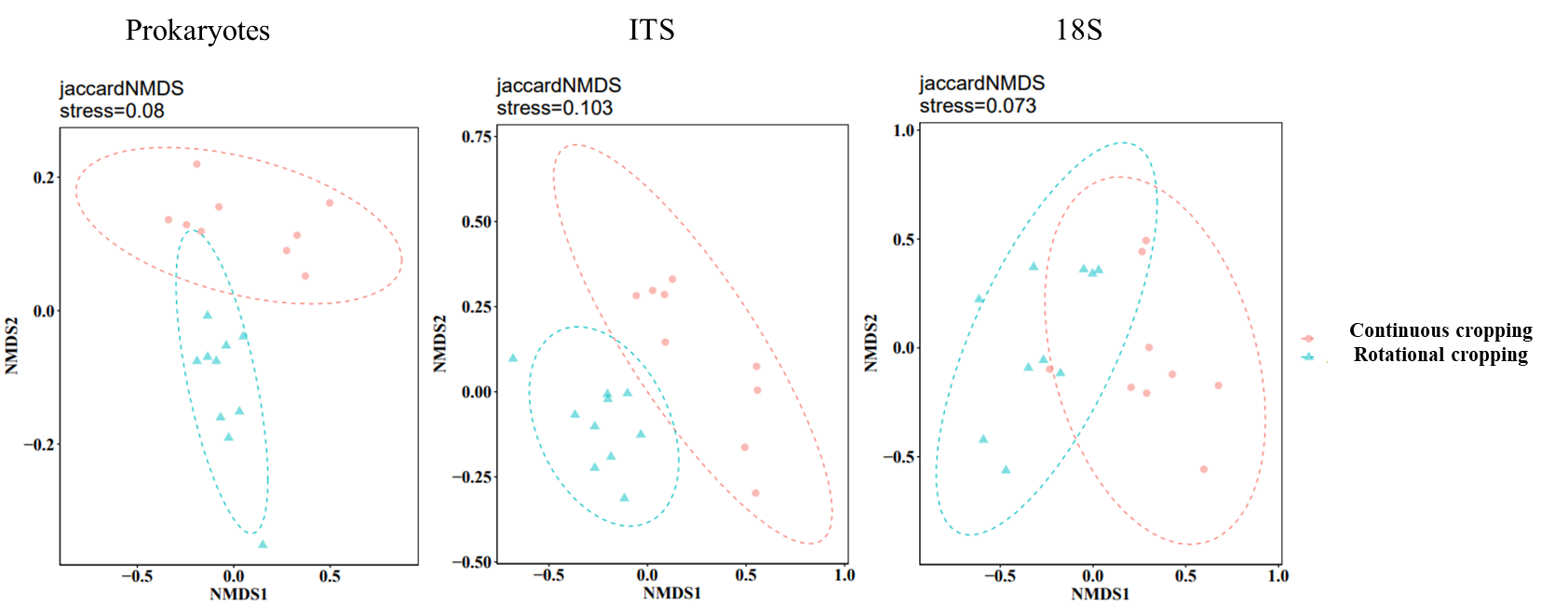


Figure S3 Relative abundance of main prokaryotes (a), fungi (b) and protist (c) phylum and genus in multiple cropping and rotational cropping. C: Continuous cropping; R: Rotational cropping


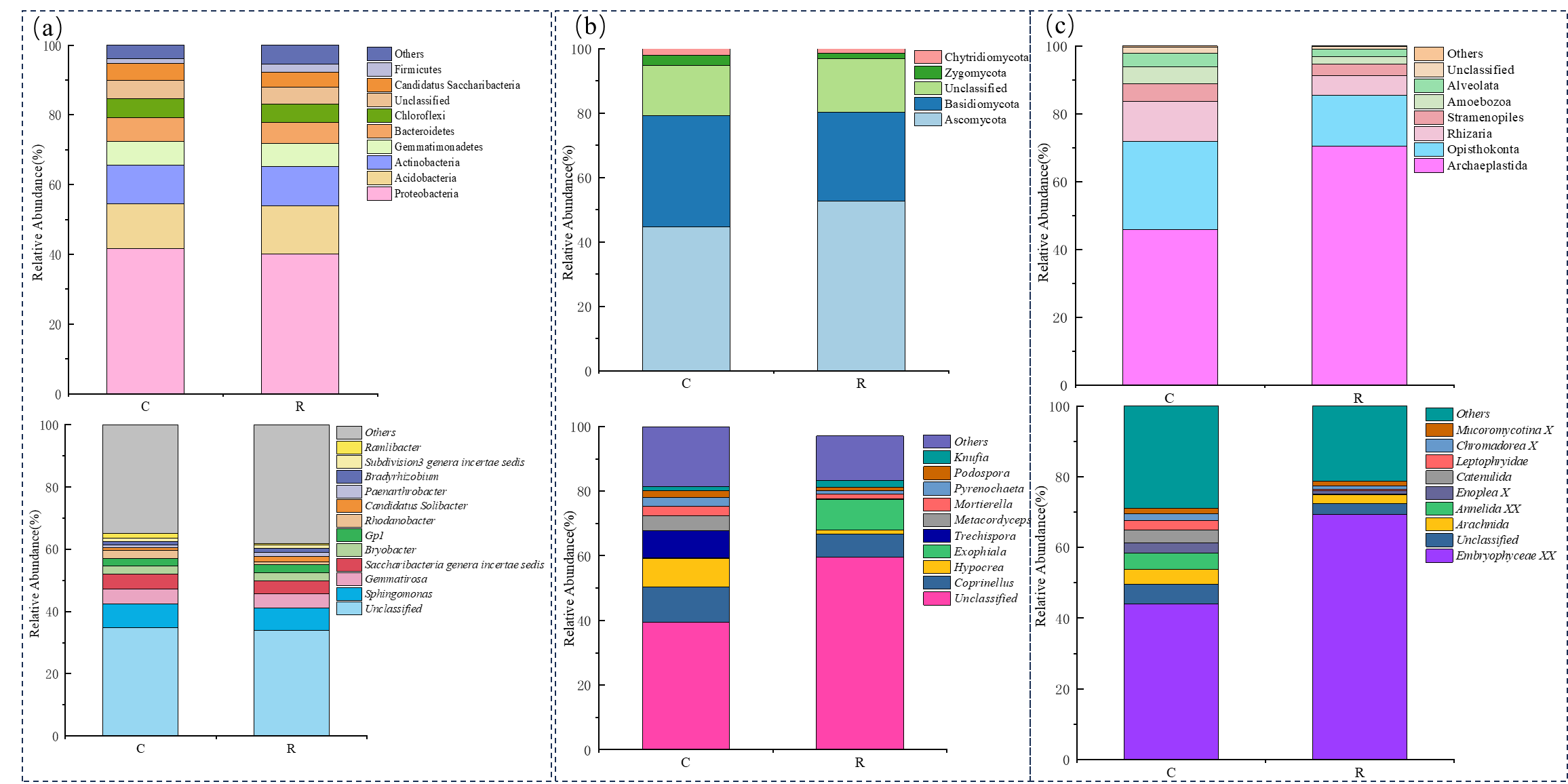

Supplement: Supplementary file 1 [file Data_Sheet_1.docx]
